# Abrikossoff Tumor (Granular Cell Tumor) Presenting in the Esophagus

**DOI:** 10.7759/cureus.13278

**Published:** 2021-02-11

**Authors:** Kevin Groudan, Jean Chalhoub, Rohit Singhania

**Affiliations:** 1 Internal Medicine, Baystate Medical Center, Springfield, USA; 2 Gastroenterology, Baystate Medical Center, Springfield, USA

**Keywords:** abrikossoff tumor, granular cell tumor, esophagus, esophagogastroduodenoscopy (egd)

## Abstract

Abrikossoff tumors, also known as granular cell tumors, are rare and often benign soft tissue neoplasms of Schwann cell origin. The vast majority of cases are reported in the skin and subcutaneous tissue. Only 0.001% of Abrikossoff tumors are estimated to occur in the esophagus. We report a rare case of Abrikossoff tumor of the esophagus in a patient who underwent esophagogastroduodenoscopy for abdominal pain and nausea.

## Introduction

First identified by Abrikossoff in 1926, granular cell tumors, or Abrikossoff tumors, are rare soft tissue neoplasms of Schwann cell origin [1]. Although they can occur anywhere in the body, Abrikossoff tumors are commonly reported in the skin and subcutaneous tissue [2]. Gastrointestinal tract Abrikossoff tumors are rare, accounting for only 6% to 10% of all Abrikossoff tumors [2]. Esophageal Abrikossoff tumors make up 30% to 60% of this subset [2]. We report a rare case of esophageal Abrikossoff tumor diagnosed in a patient who underwent esophagogastroduodenoscopy (EGD) for abdominal pain and nausea.

## Case presentation

A 48-year-old African American woman with gastroesophageal reflux disease, obesity, and pre-diabetes presented to the gastroenterology clinic reporting months of abdominal pain and nausea not responsive to daily omeprazole and as-needed calcium carbonate. She did not endorse melena, unexplained weight loss, difficulty swallowing, or a family history of cancer. She was taken for EGD to rule out upper gastrointestinal causes of her symptoms, such as peptic ulcer disease, gastritis, esophageal dysmotility, and neoplasm.

**Figure 1:**
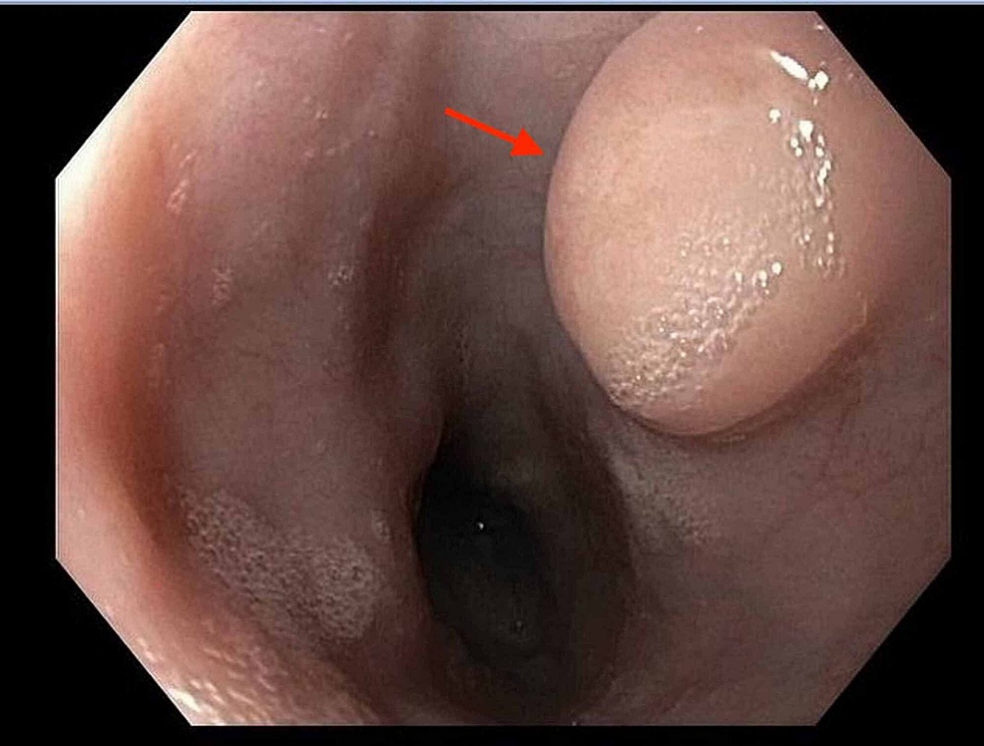
Localized, firm, mobile nodule (1 cm) with normal overlying mucosa in the upper-to-mid esophagus 25 cm from the incisors.

Her EGD was significant for a 1 cm localized, firm, mobile nodule with normal overlying mucosa in the upper-to-mid esophagus 25 cm from the incisors (Figure 1). Biopsies were obtained. Her EGD was otherwise unremarkable, and she was encouraged to continue 40 mg omeprazole daily for non-erosive reflux disease. Her biopsy results were significant for esophageal granular cell tumor (Abrikossoff tumor), confirmed by immunohistochemical staining for S100.

**Figure 2:**
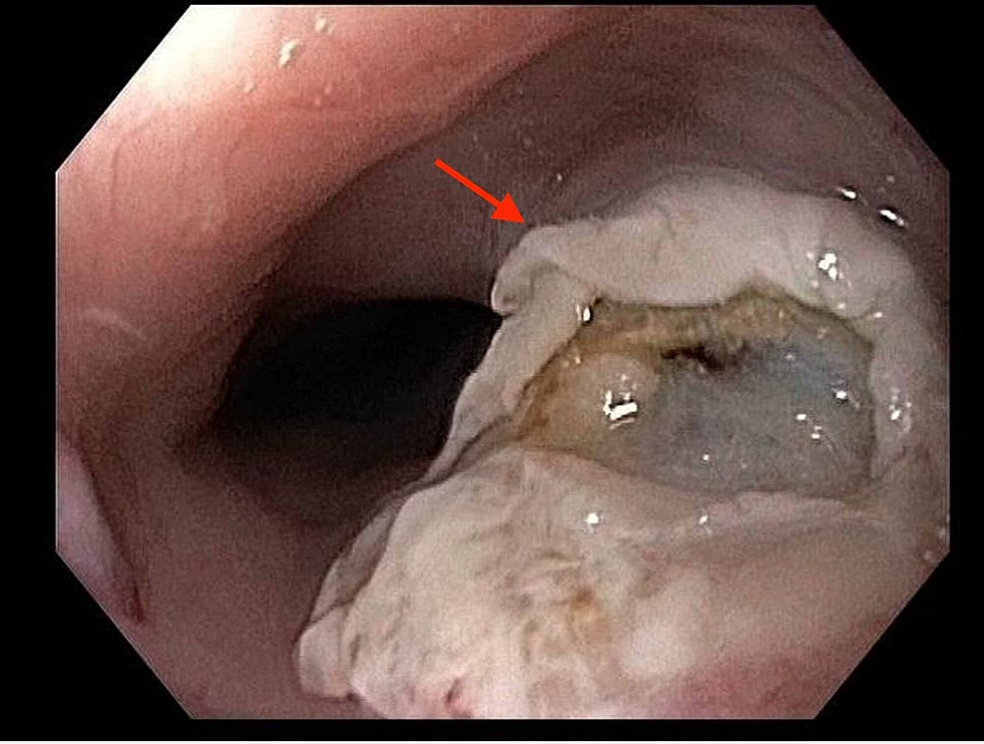
Removal of the esophageal Abrikossoff tumor by single-piece polypectomy.

She was recommended to have the tumor removed by endoscopic mucosal resection (EMR); however, due to its benign nature and uncertainty if it caused her symptoms, endoscopic surveillance every one to two years was also offered. She opted for removal and underwent repeat EGD for single-piece polypectomy (Figure 2). She was scheduled for a repeat EGD in one to two years for surveillance. Approximately six months later, she continues to have intermittent burning abdominal pain relieved with omeprazole, felt to be secondary to gastritis.

## Discussion

Approximately 0.001% of Abrikossoff tumors are estimated to occur in the esophagus [3]. The vast majority of esophageal Abrikossoff tumors are benign, with fewer than 40 malignant cases reported in the literature [3]. Although reported in all age groups, most cases occur in patients 40 to 60 years of age [4]. African Americans and women appear to be disproportionally affected [5]. Our patient met each of these demographics as she was a 48-year-old African American woman. Overall, 65% of reported cases are diagnosed in the distal esophagus, with just 15% and 20% identified in the proximal and middle esophagus, respectively [6]. Our patient’s Abrikossoff tumor was 25 cm from the incisors in her upper-to-mid esophagus. Most patients are asymptomatic at the time of diagnosis; however, symptomatic patients can present with retrosternal discomfort, epigastric pain, nausea, or vomiting [4]. Tumors larger than 2 cm in diameter are more likely to be symptomatic [4]. Our patient’s tumor was 1 cm in diameter and, in retrospect, not felt to be responsible for her symptoms.

Esophageal Abrikossoff tumors usually appear endoscopically as firm, smooth, sessile, yellow-to-gray intramural lesions with normal-appearing, non-ulcerated overlying mucosa [7]. Endoscopic ultrasound (EUS) is a useful tool for evaluating their malignancy potential by assessing tumor size, location, depth of invasion, and lymph node involvement [4]. Under EUS, they often appear as a hypoechoic, smooth-walled mass [4]. Their non-specific appearance can be confused for more common conditions, including esophageal cysts, rhabdomyomas, gastrointestinal stromal tumors, and hamartomas [4]. Biopsy and immunohistochemical staining of periodic acid-Schiff, nestin, S100, or CD68 is required for definitive diagnosis [8]. Histologically, they appear as grouped polygonal cells with small rounded nuclei and densely eosinophilic or granular cytoplasms [4]. Malignant histologic features include tumor cell necrosis and spindling, increased nuclear-to-cytoplasmic ratio, increased nucleoli size, and nuclear pleomorphism [9].

Management of esophageal Abrikossoff tumors depends on their malignancy potential. Tumors that do not cause symptoms and are less than 1 cm in diameter are typically managed conservatively with annual EGD to monitor for malignant transformation [10]. Tumors that cause symptoms, are larger than 2 cm in diameter, or have malignant features shown on EUS should be removed by EMR, endoscopic submucosal dissection (ESD), or surgery [11]. EMR is usually preferred due to its lower bleeding and perforation risk [11]. Removal by ESD reduces the risk of incomplete reaction and is preferred for larger tumors, above 1.5 cm in diameter [12]. Given that our patient’s tumor was 1 cm in diameter, it was appropriately removed by EMR. Malignant esophageal Abrikossoff tumors should be removed surgically [7]. Surgical excision of malignant tumors is often successful, with reports of only 8% tumor recurrence [7]. Radiation and chemotherapy are not recommended for the management of malignant esophageal Abrikossoff tumors [7].

## Conclusions

We present a rare case of Abrikossoff tumor of the esophagus. Given their rarity and non-specific appearance on EUS, esophageal Abrikossoff tumors can be easily mistaken for other esophageal pathologies. Evaluating their malignancy potential is key to their management. Although rare, malignant esophageal Abrikossoff tumors appear to have an excellent prognosis with surgical resection. Clinicians should be alert to the presentation, management, and prognosis of esophageal Abrikossoff tumor for prompt recognition, treatment, and improved patient outcomes.
